# Colorectal cancer outcomes show relationships with the type and extent of vascular complications in individuals with diabetes: A population‐based study

**DOI:** 10.1111/dme.70310

**Published:** 2026-03-23

**Authors:** Rebecca J. Birch, John C. Taylor, Amy Downing, Philip Quirke, Paul Finan, Katie Spencer, Eva J. A. Morris, Simon Howell, Ramzi A. Ajjan

**Affiliations:** ^1^ Pathology and Data Analytics, Leeds Institute of Medical Research at St James's University of Leeds Leeds UK; ^2^ Leeds Institute for Data Analytics University of Leeds Leeds UK; ^3^ Leeds Teaching Hospitals NHS Trust Leeds UK; ^4^ Leeds Institute of Health Sciences University of Leeds Leeds UK; ^5^ Nuffield Department of Population Health, Big Data Institute University of Oxford Oxford UK; ^6^ Leeds Institute of Cardiovascular and Metabolic Medicine University of Leeds Leeds UK

**Keywords:** admissions, cancer registry, DCSI, mortality, population‐based, surgical resection, survival

## Abstract

**Aims:**

Diabetes is associated with poorer prognosis and treatment‐related outcomes in patients with cancer. Major surgical resection is the mainstay of potentially curative treatment for colorectal cancer (CRC). This study aimed to assess whether the risk of adverse outcomes for individuals with diabetes and CRC varies by diabetes status and associated diabetes‐related complications.

**Methods:**

Information for all individuals diagnosed with CRC in England between 2011 and 2021 was obtained from cancer registry data. Pre‐existing diabetes was identified using diagnostic codes during hospital inpatient stay. Cox regression and logistic regression were used to assess the relationship between diabetes complication status and postoperative outcomes (5‐year survival, 90‐day mortality, death in hospital and unplanned readmission).

**Results:**

Of the 372,477 individuals diagnosed with CRC, treatment using major surgical resection was highest amongst those with no diabetes (60%) and diabetes without complications (62%) compared to those with combined (microvascular and macrovascular) complications (34%). Five‐year survival was lowest for those with combined complications when compared to those with no diabetes (45% vs. 69% after major resection; 5% vs. 18% without major resection). Increasing levels of complication severity were associated with increasing rates of 90‐day postoperative mortality, with combined complications associated with the poorest outcome when compared to those without diabetes (10% vs. 4%, adjusted OR 2.18, 95% CI 1.90–2.51).

**Conclusions:**

This population‐based study demonstrates that the risk of adverse outcomes in patients with diabetes and CRC is heterogeneous. Future work is required to understand whether postoperative outcomes can be improved in individuals with diabetes and CRC.

**What's new?**

- Diabetes is associated with poorer treatment‐related outcomes in patients with cancer. It is not known whether the excess mortality risk in patients with colorectal cancer is heterogeneous or if those experiencing microvascular and/or macrovascular complications have different risk profiles.
- Individuals with diabetes complications underwent less potentially curative major surgery than those without diabetes or complications. Increasing levels of complication severity were associated with poorer postoperative outcomes, including mortality.
- Further work is required to understand whether outcomes in the highest risk groups can be improved and whether early involvement of diabetes teams in patient management is required.

## 1 INTRODUCTION

Up to a tenth of the population of England are thought to have diabetes, including 7% with an official diagnosis and 3% undiagnosed.^1^ Studies have shown diabetes to be associated with an increased risk of colorectal cancer (CRC),^2^ and individuals with diabetes who do develop cancer have a worse prognosis than their peers without diabetes.^3^ In particular, diabetes is associated with worse treatment‐related outcomes in patients with cancer^4^ and is an independent predictor of mortality in those with CRC.^5^ Studies outside of the cancer setting have consistently shown that diabetes is associated with inferior surgical outcomes but the exact contributory factor(s) remain largely unknown.^6^ As major surgical resection is the mainstay of potentially curative treatment for CRC, the risk for individuals with diabetes, constituting up to 15% of this population,^7^ requires investigation.

Diabetes complications are broadly divided into microvascular, including retinopathy, nephropathy and neuropathy, whereas macrovascular complications include cardiovascular, cerebrovascular and peripheral vascular disease.^8^ Previous work suggests that the excess risk of mortality in diabetes is heterogeneous and related, at least in part, to the presence of vascular complications.^9^ However, studies have generally failed to consider the differential effect of the various diabetic complications, and this remains an incompletely studied area, particularly in individuals with cancer. This is of clinical importance as limited data show that the relationship between diabetic complications and survival is not uniform; for example, a higher proportion of deaths have been reported in those with macrovascular than microvascular complications.^10^

Accurate risk assessment in a cancer population is vital to ensure that patients are not under‐treated or indeed exposed to unacceptable levels of risk. To effectively risk stratify patients with other comorbidities and diabetes, it is important to investigate whether all complications carry the same weight. We hypothesise that the risk of adverse outcomes in patients with diabetes and CRC varies according to the type of associated vascular complication. Therefore, the aim of this work was to determine whether, in patients with CRC, the risk associated with diabetes complications is equal across all groups or whether the different groups (experiencing microvascular and/or macrovascular complications) have different risk profiles, thus allowing better stratification and treatment of patients with the potential to improve short‐ and long‐term clinical outcomes.

## 2 METHODS

### 2.1 Study population and data sources

Information was extracted for all individuals diagnosed with a first primary CRC (ICD‐10 C18‐C20) in England between 1st January 2011 and 31st December 2021 from the COloRECTal cancer data Repository (CORECT‐R) which holds information on all cases of CRC diagnosed and treated in the English NHS.^11^ Information on age at diagnosis, sex, stage of disease, tumour site, survival time and socioeconomic status (based on the income domain of the Index of Multiple Deprivation (IMD) 2019) was obtained from the National Cancer Registration and Analysis Service (NCRAS) component of this resource. Details of the surgical management of CRC were obtained from the Hospital Episode Statistics (HES) component.

Major surgical resections were grouped using predefined methodology.^12^ If individuals were not determined to have undergone a major surgical resection, they were assigned to the ‘no major resection’ group, which included those with palliative procedures and those who underwent minor resections and biopsies. Ninety‐day postoperative mortality was defined using the date of major surgical resection and date of death. Death in hospital was recorded if an individual died during the same hospital admission as their major surgical resection. Unplanned readmission was an emergency admission to hospital within the 30 days following discharge from the admission during which major surgical resection was undertaken. Postoperative length of inpatient stay was calculated using the date of major surgical resection and date of discharge from hospital, or death. This includes in hospital transfers (between wards or units) but will exclude transfers to other hospitals or healthcare units which would be classified as a separate admission using this methodology. Prolonged length of stay (LOS) was defined as a stay lasting 21 days or more following surgery.^13^

### 2.2 Diabetes status

Diabetes status was determined from HES data. Individuals were classified as having pre‐existing diabetes if the relevant ICD‐10 codes (E10‐E14) had been reported during an inpatient stay within the 6 years preceding the CRC diagnosis. Six years has been demonstrated in other studies to be the optimal period for identifying pre‐existing comorbidities in routinely collected healthcare data.^14^ Complication status was determined using the adapted Diabetes Complications Severity Index (aDCSI).^15^

Patients were assigned to one of five categories based on their diabetes complication status defined using ICD‐10 codes reported during inpatient admissions; (i) no diabetes, (ii) diabetes with no complications, (iii) diabetes with microvascular complications, (iv) diabetes with macrovascular complications or (v) diabetes with combined microvascular and macrovascular complications.^8^ Individuals were assigned to the no complications group if they were identified as having diabetes but did not have any of the additional, relevant ICD‐10 codes recorded (Table S1). Those who were classified as having microvascular complications had ocular, renal or neurological complications identified; those with macrovascular complications had cerebrovascular, cardiovascular or peripheral vascular complications identified (Table S1).

### 2.3 Statistical analysis

Descriptive analyses were performed to assess any variation in the characteristics between the complication groups. An intersection plot was produced using UpSet^16^ in R to visualise and describe the groupings of complications within those who were identified as having diabetes‐related complications. The plot shows the number of individuals, with diabetes, in each group (presented as the intersection size), with each combination of diabetes‐related complications. It also presents the number of individuals with each complication in isolation (presented as the set size). It is important to note that individuals can be present in multiple sets but only one intersection.

Survival time was calculated from the date of CRC diagnosis to the date of death or censoring (31st March 2024) for individuals who did not undergo a major surgical resection. For those who had undergone surgery, survival time was calculated from the date of major surgical resection to death or censoring. The survival of the two groups (those who underwent a major resection and those who did not) is not directly comparable as the survival time is measured from different origins. Multivariable Cox regression models and Kaplan–Meier curves were used to assess the relationship between diabetes complication status and 5‐year survival. Models were adjusted for baseline patient characteristics (age, sex and socioeconomic status) and tumour characteristics (site, stage of disease and year of diagnosis), which were selected due to their known relationship with CRC and diabetes outcomes. Plots of scaled Schoenfield residuals against time and log–log plots of the survival probabilities versus log(time) were used check there was no violation in the assumption of proportionality for the cox regression models.

Multivariable logistic regression was used to assess any relationship between diabetes complications status and postoperative outcomes following major resection (90‐day postoperative mortality, death in hospital, unplanned readmission and prolonged LOS). Models were adjusted for age at diagnosis, sex, socioeconomic deprivation, tumour site, urgency of surgery and year of cancer diagnosis.

## 3 RESULTS

### 3.1 Study population

Of the 372,477 individuals diagnosed, 43,777 (11.8%) were recorded as having diabetes mellitus. Within the study population, 4838 (11.1%) had microvascular complications, 13,727 (31.4%) had macrovascular complications and 7779 (17.8%) had combined (both microvascular and macrovascular) complications. Close to 40% of individuals with diabetes (*n* = 17,433, 39.8%) had no complications identified (Table 1).

**TABLE 1:** Characteristics of the study population by complication group.

	No diabetes	Diabetes – no complications	Diabetes – microvascular complications	Diabetes – macrovascular complications	Diabetes – combined complications
	*n* (%)	*n* (%)	*n* (%)	*n* (%)	*n* (%)
Age at diagnosis					
<50	20,046 (6.1)	389 (2.2)	56 (1.2)	56 (0.4)	24 (0.3)
50–59	38,727 (11.8)	1522 (8.7)	235 (4.9)	516 (3.8)	157 (2)
60–69	79,283 (24.1)	4313 (24.7)	767 (15.9)	2420 (17.6)	864 (11.1)
70–79	99,038 (30.1)	6412 (36.8)	1697 (35.1)	5264 (38.3)	2562 (32.9)
≥80	91,606 (27.9)	4797 (27.5)	2083 (43.1)	5471 (39.9)	4172 (53.6)
Sex					
Female	147,956 (45)	7263 (41.7)	2167 (44.8)	4450 (32.4)	2804 (36)
Male	180,744 (55)	10,170 (58.3)	2671 (55.2)	9277 (67.6)	4975 (64)
Socioeconomic status					
1 – Most affluent	73,431 (22.3)	3046 (17.5)	772 (16)	2328 (17)	1222 (15.7)
2	76,079 (23.1)	3615 (20.7)	1016 (21)	2679 (19.5)	1545 (19.9)
3	68,863 (21)	3566 (20.5)	980 (20.3)	2949 (21.5)	1657 (21.3)
4	59,021 (18)	3637 (20.9)	1058 (21.9)	2812 (20.5)	1636 (21)
5 – Most deprived	51,306 (15.6)	3569 (20.5)	1012 (20.9)	2959 (21.6)	1719 (22.1)
Tumour stage					
I	50,488 (15.4)	2907 (16.7)	717 (14.8)	2420 (17.6)	1185 (15.2)
II	75,937 (23.1)	4265 (24.5)	1081 (22.3)	3066 (22.3)	1538 (19.8)
III	89,303 (27.2)	4698 (26.9)	1196 (24.7)	3173 (23.1)	1577 (20.3)
IV	72,585 (22.1)	3508 (20.1)	1050 (21.7)	2600 (18.9)	1472 (18.9)
Unknown	40,387 (12.3)	2055 (11.8)	794 (16.4)	2468 (18)	2007 (25.8)
Tumour site					
Right colon	113,574 (34.6)	6741 (38.7)	2007 (41.5)	5510 (40.1)	3263 (41.9)
Left colon	91,081 (27.7)	4796 (27.5)	1249 (25.8)	3751 (27.3)	1953 (25.1)
Colon unspecified	13,529 (4.1)	685 (3.9)	216 (4.5)	685 (5)	574 (7.4)
Rectosigmoid	19,241 (5.9)	940 (5.4)	261 (5.4)	689 (5)	384 (4.9)
Rectum	91,275 (27.8)	4271 (24.5)	1105 (22.8)	3092 (22.5)	1605 (20.6)
Surgical management					
Major resection	197,867 (60.2)	10,756 (61.7)	2509 (51.9)	6641 (48.4)	2646 (34)
Minor resection	22,198 (6.8)	1262 (7.2)	351 (7.3)	1188 (8.7)	616 (7.9)
Palliative surgery	18,786 (5.7)	870 (5)	274 (5.7)	728 (5.3)	411 (5.3)
No NHS surgery	89,849 (27.3)	4545 (26.1)	1704 (35.2)	5170 (37.7)	4106 (52.8)
Type of complication^a^					
Ocular			2791 (57.7)		3311 (42.6)
Neurological			375 (7.8)		957 (12.3)
Renal			2371 (49)		5480 (70.4)
Cerebrovascular				2019 (14.7)	1210 (15.6)
Cardiovascular				12,509 (91.1)	7122 (91.6)
Peripheral vascular				1453 (10.6)	1535 (19.7)
	328,700	17,433	4838	13,727	7779

^a^ Individuals may be present in multiple complication categories.

### 3.2 Characteristics

Patient characteristics varied between the groups (no diabetes, no complications, microvascular, macrovascular or combined). A higher proportion of patients with combined complications were aged ≥80 at the time of their CRC diagnosis, than those in any other group (Table 1). Those with no complications and no diabetes were significantly younger than any group with complications. A higher proportion of those with diabetes lived in the most socioeconomically deprived areas; this increased from 20.5% (*n* = 3569) of those with no complications to 22.1% (*n* = 1719) of those with combined microvascular and macrovascular complications, compared to 15.6% of those without diabetes. Tumours of the right colon were seen more frequently amongst those with diabetes than those without, whereas tumours of the rectum were less frequently observed.

The proportion of patients who underwent a major surgical resection for their CRC was highest amongst those with no diabetes and diabetes without complications (*n* = 197,867, 60.2% and *n* = 10,756, 61.7% respectively). In contrast, only 34.0% of those with combined complications underwent a major surgical resection (Table 1).

### 3.3 Complication types

Cardiovascular complications (alone or in combination) were the most frequently identified amongst those with diabetes (*n* = 19,631, 44.8%), followed by renal (*n* = 7851, 17.9%) or ocular (*n* = 6102, 13.9%) complications (Table 1). Complications often did not occur in isolation, instead forming clusters with other complications (Table S2 and Figure S1).

### 3.4 Outcomes in those who underwent major surgical resection

**FIGURE 1:**
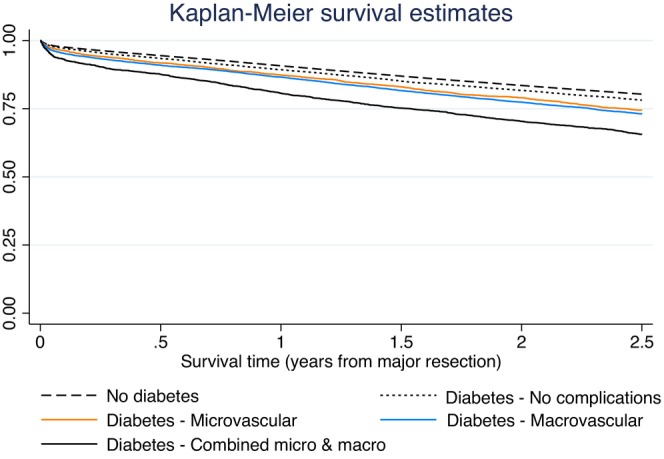
Survival from operation date for individuals with colorectal cancer who underwent a major surgical resection, by diabetes status and diabetic complication group.

Amongst patients who underwent a major surgical resection, reduced survival was noted in those with microvascular complications. Compared to individuals without diabetes, where 68.5% of individuals survived to at least 5 years from surgery, and individuals with diabetes but no complications (64.8%), a lower proportion of individuals survived a minimum of 5 years from major surgical resection (57.3%). Survival was reduced further in the presence of macrovascular complications (55.6% alive at 5 years), with the lowest survival observed in those with combined complications (45.1%) alive at 5 years (Figure 1).

Following adjustment for patient factors (age, sex and socioeconomic status) and tumour characteristics (year of CRC diagnosis, stage of disease and tumour site), the presence of diabetes, with or without complications was associated with reduced survival amongst individuals who underwent a major surgical resection of their CRC tumour. Amongst those with complications hazard ratios were lowest in the presence of microvascular complications only (HR 1.36, 95% CI 1.29–1.43) and highest in individuals with combined complications (HR 1.90, 95% CI 1.81–1.99) (Table 2).

**TABLE 2:** Multivariable Cox regression model results in relation to aDCSI status, undertaken separately according to surgical treatment status.

	No major surgical resection^a^	*p*‐value	Major surgical resection^b^	*p*‐value
	HR (95% CI)		HR (95% CI)	
aDCSI group				
No diabetes	1.00 (reference)		1.00 (reference)	
No complications	0.97 (0.94–1.00)	0.031	1.19 (1.15–1.22)	<0.001
Microvascular only	1.08 (1.03–1.13)	<0.001	1.36 (1.29–1.43)	<0.001
Macrovascular only	1.11 (1.08–1.14)	<0.001	1.48 (1.43–1.53)	<0.001
Combined	1.28 (1.24–1.32)	<0.001	1.90 (1.81–1.99)	<0.001
Age at diagnosis				
<50	1.00 (reference)		1.00 (reference)	
50–59	1.16 (1.12–1.20)	<0.001	1.07 (1.03–1.11)	0.001
60–69	1.35 (1.31–1.40)	<0.001	1.28 (1.24–1.33)	<0.001
70–79	2.02 (1.96–2.09)	<0.001	2.15 (2.08–2.22)	<0.001
≥80	3.24 (3.14–3.34)	<0.001	3.96 (3.83–4.10)	<0.001
Sex				
Male	1.00 (reference)		1.00 (reference)	
Female	1.03 (1.02–1.04)	<0.001	0.86 (0.84–0.87)	<0.001
Socioeconomic status				
1 – Most affluent	1.00 (reference)		1.00 (reference)	
2	1.10 (1.08–1.12)	<0.001	1.06 (1.04–1.09)	<0.001
3	1.16 (1.14–1.18)	<0.001	1.11 (1.09–1.14)	<0.001
4	1.23 (1.20–1.25)	<0.001	1.21 (1.18–1.23)	<0.001
5 – Most deprived	1.34 (1.31–1.36)	<0.001	1.34 (1.31–1.37)	<0.001
Tumour site				
Right colon	1.00 (reference)		1.00 (reference)	
Left colon	0.79 (0.78–0.80)	<0.001	0.87 (0.86–0.89)	<0.001
Colon, unspecified	1.23 (1.20–1.26)	<0.001	1.07 (1.02–1.13)	0.006
Rectosigmoid	0.88 (0.86–0.90)	<0.001	0.88 (0.85–0.90)	<0.001
Rectum	0.67 (0.66–0.68)	<0.001	0.89 (0.88–0.91)	<0.001
Tumour stage				
I	1.00 (reference)		1.00 (reference)	
II	2.30 (2.24–2.37)	<0.001	1.37 (1.34–1.40)	<0.001
III	2.74 (2.67–2.81)	<0.001	2.16 (2.11–2.21)	<0.001
IV	7.14 (6.98–7.31)	<0.001	6.77 (6.59–6.95)	<0.001
Unknown	3.36 (3.28–3.45)	<0.001	2.00 (1.92–2.09)	<0.001
Year of CRC diagnosis	1.00 (0.99–1.00)	<0.001	0.97 (0.97–0.98)	
Surgical urgency				
Elective			1.00 (reference)	
Emergency			1.96 (1.93–1.99)	<0.001

^a^ Survival time calculated from diagnosis date.

^b^ Survival time calculated from operation date.

**FIGURE 2:**
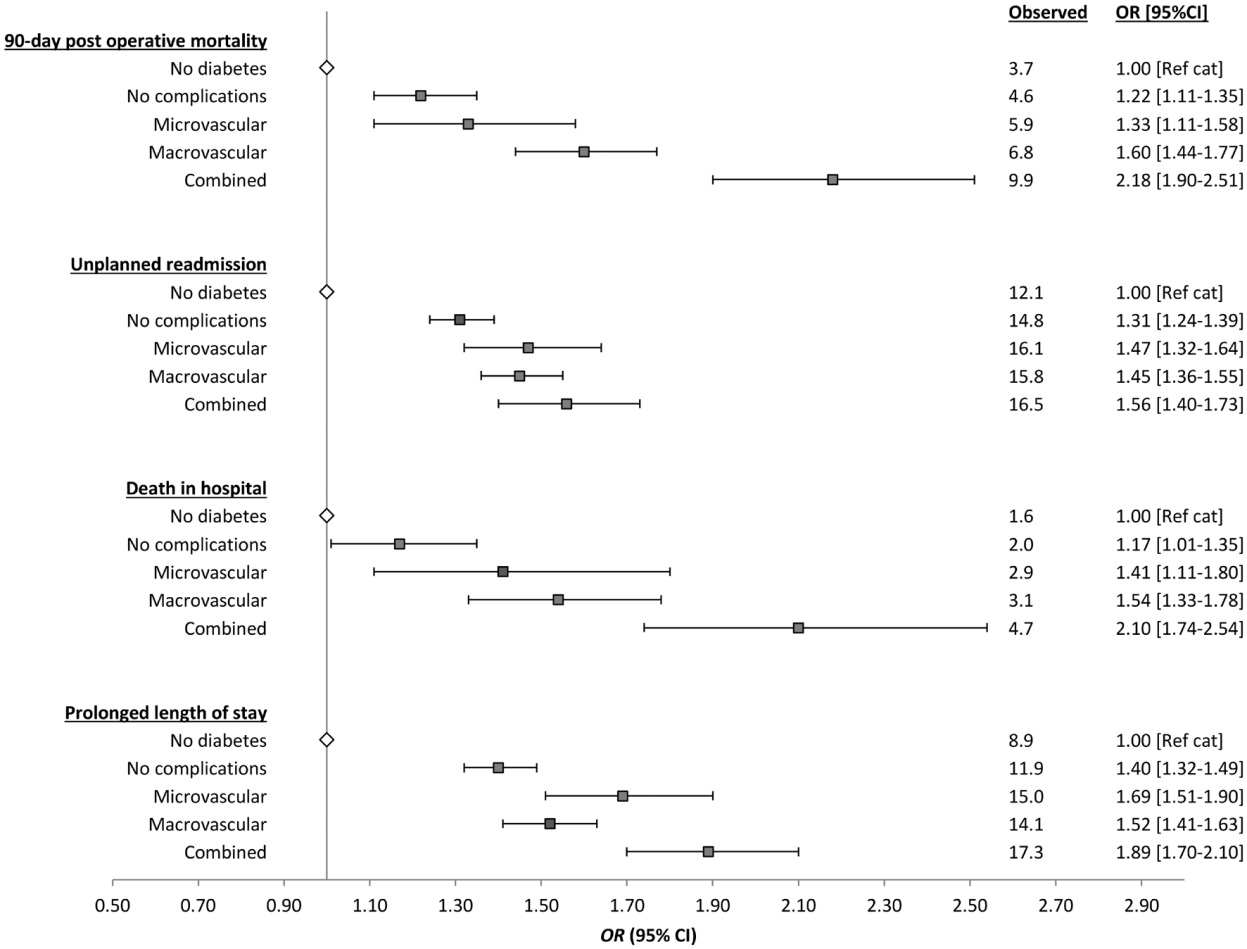
Results of adjusted logistic regression models in relation to aDCSI status. Each outcome modelled separately (full results of adjusted models available Tables S3–S6).

The presence of diabetes‐related complications was associated with significantly higher rates of poor immediate postoperative outcomes, 90‐day postoperative mortality, unplanned readmission to hospital, death within the same admission as surgery and prolonged LOS (Figure 2 and Tables S3–S6). Increasing levels of complication severity were associated with increasing rates of 90‐day postoperative mortality, with the presence of combined complications associated with the poorest outcomes when compared to those without diabetes (OR 2.18, 95% CI 1.90–2.51 for individuals with combined complications compared to those without diabetes). The rate of unplanned readmissions to hospital following discharge after surgery also increased with increasing levels of diabetes complications. Compared to individuals without diabetes, the OR for readmission was 1.31 (95% CI 1.24–1.39) for those with diabetes and no complications, increasing to OR 1.56 (95% CI 1.40–1.73) for those with combined complications. Similarly, increasing levels of complication severity were associated with death during the same hospital stay, as individuals with combined complications were twice as likely to die during their hospital stay following major surgery than those without diabetes (OR 2.10, 95% CI 1.74–2.54). Increasing rates of prolonged LOS were also more likely for those with diabetes and complications than for those without diabetes.

### 3.5 Outcomes in those who did not undergo major surgical resection

**FIGURE 3:**
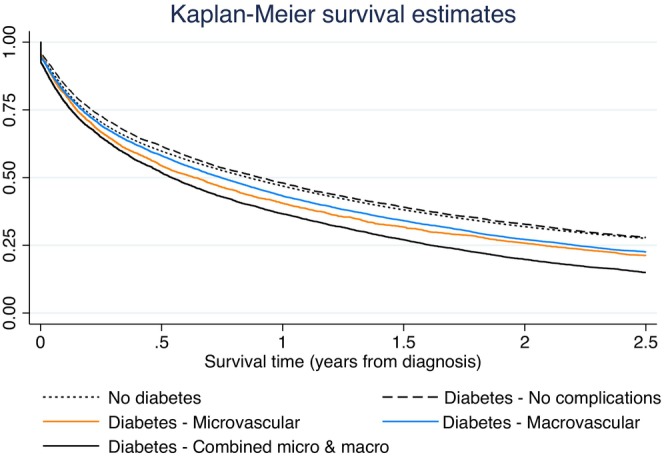
Survival from diagnosis date for individuals with colorectal cancer who did not undergo a major surgical resection, by diabetes status and diabetic complication group.

At 5 years from CRC diagnosis, 17.4% of those with diabetes but no complications were alive, compared to 18.4% of those without diabetes. The presence of combined complications was associated with the lowest survival of 5.2%, versus 11.0% in those with microvascular complications alone and 11.3% in those with macrovascular complications alone (Figure 3).

The presence of diabetes‐related complications was associated with significantly worse survival, compared to those without diabetes; microvascular complications alone, HR 1.08 (95% CI 1.03–1.13), macrovascular alone HR 1.11 (95% CI 1.08–1.14), combined complication HR 1.28 (95% CI 1.24–1.32) (Table 2). In contrast, those with diabetes but no complications had no increase in death compared to individuals without diabetes (HR 0.97, 95% CI 0.94–1.00).

## 4 DISCUSSION

Whilst the relationship between diabetes and cancer outcomes has been well‐described in the literature,^5^ limited studies have stratified the diabetes population in relation to their overall complication status^9^ and no work has analysed the relative importance of specific subtypes of complications at a population level. While findings of worsening life expectancy in the presence of macrovascular complications have been reported in the diabetes literature,^17^, ^18^, ^19^ our study is the first, to our knowledge, to investigate the association of micro‐ and macrovascular diabetes complications separately and in combination with outcomes in a cancer population, using real‐world unselected healthcare data. Our work demonstrates that the impact of diabetes on CRC outcomes is heterogeneous and appears to be influenced by the presence of diabetes complications.

Survival, both short and longer term, as well as length of hospital stay are affected by diabetes complications with little effect for diabetes per se. In general, clinical outcomes can be divided into five categories with the best observed in those without diabetes, followed closely by those with diabetes and no documented complications. However, once diabetes complications are clinically evident, prognosis worsens in a stepwise manner with the presence of microvascular complications being the first step followed by macrovascular complications, while the combination of the two resulting in the worst prognosis. These patterns were consistent regardless of whether individuals received potentially curative or non‐curative therapies and the findings were unchanged following adjustments for potential confounding factors. It is important to note that due to the differing origin points used to calculate survival for those who underwent major surgical resection and those who did not, the survival should not be compared across treatment groups.

Diabetes had a larger effect on overall survival in the presence of complications, indicating that our understanding of the role of diabetes on adverse outcomes in CRC patients’ needs to be refined. More specifically, subcategories of patients are required, defined according to the type and seriousness of diabetes complications as well as the number of complications. This study assessed all‐cause mortality, meaning that deaths due to any cause were included in the analysis. The data utilised do not include cause of death information, meaning it was not possible to distinguish between cancer and other causes. It is known that certain diabetes‐related complications are associated with higher death rates in the general population and so further work is needed to address the competing risks in this population.

Cardiovascular disease was the commonest type of diabetes‐related complication followed by nephropathy while neuropathy was the least common. While the absence of complications in the diabetes population was associated with improved clinical outcomes in general, some adverse effects were still evident. For example, early 90‐day mortality following surgical intervention was affected by the presence of diabetes, even in those without complications. There is a need to better understand the reasons underpinning this finding, whether related to unobserved patient or indeed healthcare‐related factors. Moreover, length of hospital stay and hospital readmissions were affected by diabetes alone, while the presence of complications had a relatively modest additional effect, supporting the need for better understanding of the causal pathways involved. One important factor to consider is glycaemic control, as higher blood glucose levels are associated with increased diabetes‐related complications in the long term.^20^ Whilst these complications are likely to be captured by the aDCSI measure, acute fluctuations in blood glucose levels may occur in individuals without occurrence of the complications listed. The data required to examine the association between glycaemic control and outcomes was not available for this study. Future research is warranted to investigate this and uncover other important factors, as well as understanding whether these are modifiable, and could thus improve short‐term outcomes and reduce hospital stay.

This study has a number of strengths including the inclusion of a large population and analysis according to diabetes complication load, while making appropriate adjustments for confounders. However, there are weaknesses to be acknowledged. First, both the diabetes and complication status of individuals were derived from secondary care data, which could lead to an under capture of individuals managed solely by primary care. In this study, the proportion of individuals with diabetes and those who were identified as having diabetes‐related complications using the aDCSI measure generally reflects what has been observed in the diabetes population.^21^ However, studies have also suggested that the use of secondary care data alone may lead to significant under capture of diabetes status,^22^, ^23^ highlighting the need for caution. If there was under capture of individuals with diabetes managed solely by primary care, the fact that they have not required any admission to hospital in the previous 6 years suggests that they are unlikely to have any other long‐term health conditions and their diabetes is adequately controlled and might well be expected to be uncomplicated as a result. Second, the identification of diabetes‐related complications is similarly influenced by the data source used, as these are recorded during an inpatient admission in the 6 years preceding the CRC diagnosis. For severe complications such as cardiovascular disease, the impact is likely to be minimal as these would routinely precipitate inpatient care and so are at increased odds of being recorded during a hospital stay. Consequently, this study is likely capturing the impact of advanced complications and further work, using linked primary care data, will clarify whether early complications have an impact. Third, it can be argued that patient characteristics differed between the different subgroups with complications. Individuals in the combined microvascular and macrovascular complications group were older and came from areas with increased socioeconomic deprivation, while also having lower rates of major surgical resection. However, our findings remained consistent after adjusting for these factors. The subgroups may differ in other ways for which data are not readily available, such as exercise levels or health literacy, and it is possible that these unmeasured factors are contributing to a difference in outcomes. Additionally, the coding system used does not have the capacity to differentiate between type 1 and type 2 diabetes, and therefore it was not possible to examine the relationships between type of diabetes, complications and outcomes. Variables such as frailty, BMI and functional status were not available in the routinely collected data used in this project, meaning that it was not possible to adjust for their impact on postoperative outcomes. Further work is required to investigate the relationship between diabetes and postoperative outcomes in the context of additional patient details, focusing on their relationship with diabetes status.

The results of this study have implications for clinical practice, as it suggests that the additional risk associated with diabetes in the CRC population may not be homogeneous and that further work is needed to understand the highest risk groups. This may lead to adoption of a multidisciplinary approach to care in the highest risk group, including early involvement of the diabetes teams in patient management, which may improve survival as it has been shown to have the potential to improve outcomes for individuals with diabetes who are admitted to hospital in other specialities.^24^, ^25^, ^26^

In conclusion, this study demonstrates that the risk of adverse outcomes in patients with diabetes and CRC is heterogeneous. Reduction in overall survival is influenced by the presence of diabetes complications and related to complication load, with the highest risk observed in those with both microvascular and macrovascular disease. In contrast, diabetes itself appears to affect length of hospital stay and unplanned readmission and the presence of complications has a limited effect. Future work is required to understand how survival, length of stay and hospital readmissions can be improved in individuals with diabetes and CRC.

## AUTHOR CONTRIBUTIONS

R.J.B., J.C.T., A.D., P.Q., P.F., K.S., E.J.A.M., S.H. and R.A.A. were involved in the conception, design and conduct of the study and the analysis and interpretation of the results. R.J.B. wrote the first draft of the manuscript, and all authors edited, reviewed and approved the final version of the manuscript. R.J.B. and J.C.T. are the guarantors of this work and, as such, had full access to all the data in the study and take responsibility for the integrity of the data and the accuracy of the data analysis.

## CONFLICT OF INTEREST STATEMENT

The authors declare no conflicts of interest.

## Supporting information


**Table S1.** Complications status and groupings (based on the aDCSI measure).
**Table S2**. Frequency of diabetes‐related complication groupings (*counts <5 are suppressed).
**Table S3**. Results of multivariable logistic regression for 90‐day post‐operative mortality, restricted to individuals who underwent a major surgical resection.
**Table S4**. Results of multivariable logistic regression for unplanned readmission to hospital, restricted to individuals who underwent a major surgical resection.
**Table S5**. Results of multivariable logistic regression for death in hospital, restricted to individuals who underwent a major surgical resection.
**Table S6**. Results of multivariable logistic regression for prolonged length of hospital stay, restricted to individuals who underwent a major surgical resection.
**Figure S1**. Frequency of diabetes‐related complications (bars represent the number of individuals with diabetes in each subset) Intersects with a group size of ≥50 are presented.

## Data Availability

The data that support the findings of this study are available from CORECT‐R via the UK Colorectal Cancer Intelligence Hub but restrictions apply to the availability of these data, which were used under license for the current study and therefore are not publicly available.
