# Supplementary material for: Colorectal cancer outcomes show relationships with the type and extent of vascular complications in individuals with diabetes: A population‐based study
Source: Diabet Med. 2026 Mar 23;43(8):e70310. doi: 10.1111/dme.70310 (PMC13380378; doi:10.1111/dme.70310)
Supplement: Supplementary file 1 — Table S1. Complications status and groupings (based on the aDCSI measure). Table S2. Frequency of diabetes‐related complication groupings (*counts <5 are suppressed). Table S3. Results of multivariable logistic regression for 90‐day post‐operative mortality, restricted to individuals who underwent a major surgical resection. Table S4. Results of multivariable logistic regression for unplanned readmission to hospital, restricted to individuals who underwent a major surgical resection. Table S5. Results of multivariable logistic regression for death in hospital, restricted to individuals who underwent a major surgical resection. Table S6. Results of multivariable logistic regression for prolonged length of hospital stay, restricted to individuals who underwent a major surgical resection. Figure S1. Frequency of diabetes‐related complications (bars represent the number of individuals with diabetes in each subset) Intersects with a group size of ≥50 are presented. [file DME-43-e70310-s001.docx]

**Supplementary Information**

**Colorectal cancer outcomes show relationships with the type and extent of vascular complications in individuals with diabetes**

Rebecca J Birch, John Taylor, Amy Downing, Philip Quirke, Paul Finan, Katie Spencer, Eva J A Morris, Simon Howell, Ramzi A. Ajjan

**Supplementary table 1**: Complications status and groupings (based on the aDCSI measure)

| ICD10 code | Description | Uncomplicated | Complicated | Complication category |  |
| --- | --- | --- | --- | --- | --- |
| E109 | Insulin-dependent diabetes mellitus without complication |  |  |  |  |
| E119 | Non-insulin-dependent diabetes mellitus without complications |  |  |  |  |
| E129 | Malnutrition-related diabetes mellitus without complications |  |  |  |  |
| E139 | Other specified diabetes mellitus without complications |  |  |  |  |
| E149 | Unspecified diabetes mellitus without complications |  |  |  |  |
| E103 | Insulin-dependent diabetes mellitus with ophthalmic complications |  |  | Ocular | MICROVASCULAR COMPLICATIONS |
| E113 | Non-insulin-dependent diabetes mellitus with ophthalmic complications |  |  |  |  |
| E123 | Malnutrition-related diabetes mellitus with ophthalmic complications |  |  |  |  |
| E133 | Other specified diabetes mellitus with ophthalmic complications |  |  |  |  |
| E143 | Unspecified diabetes mellitus with ophthalmic complications |  |  |  |  |
| H280 | Diabetic cataract |  |  |  |  |
| H350 | Background retinopathy and retinal vascular changes |  |  |  |  |
| H353 | Degeneration of macula and posterior pole |  |  |  |  |
| H356 | Retinal haemorrhage |  |  |  |  |
| H358 | Other specified retinal disorders |  |  |  |  |
| H360 | Diabetic retinopathy |  |  |  |  |
| H352 | Other proliferative retinopathy |  |  |  |  |
| H330 | Retinal detachment and breaks |  |  |  |  |
| H540 | Blindness, binocular |  |  |  |  |
| H544 | Blindness, monocular |  |  |  |  |
| H431 | Vitreous haemorrhage |  |  |  |  |
| E102 | Insulin-dependent diabetes mellitus with renal complications |  |  | Renal |  |
| E112 | Non-insulin-dependent diabetes mellitus with renal complications |  |  |  |  |
| E122 | Malnutrition-related diabetes mellitus with renal complications |  |  |  |  |
| E132 | Other specified diabetes mellitus with renal complications |  |  |  |  |
| E142 | Unspecified diabetes mellitus with renal complications |  |  |  |  |
| N083 | Glomerular disorders in diabetes mellitus |  |  |  |  |
| N03 | Chronic nephritic syndrome |  |  |  |  |
| N04 | Nephrotic syndrome |  |  |  |  |
| N05 | Unspecified nephritic syndrome |  |  |  |  |
| N17 | Acute renal failure |  |  |  |  |
| N18 | Chronic kidney disease |  |  |  |  |
| N19 | Unspecified kidney failure |  |  |  |  |
| T824 | Mechanical complication of vascular dialysis catheter |  |  |  |  |
| Z49 | Care involving dialysis |  |  |  |  |
| Z992 | Dependence on renal dialysis |  |  |  |  |
| E104 | Insulin-dependent diabetes mellitus with neurological complications |  |  | Neurological |  |
| E114 | Non-insulin-dependent diabetes mellitus with neurological complications |  |  |  |  |
| E124 | Malnutrition-related diabetes mellitus with neurological complications |  |  |  |  |
| E134 | Other specified diabetes mellitus with neurological complications |  |  |  |  |
| E144 | Unspecified diabetes mellitus with neurological complications |  |  |  |  |
| G609 | Hereditary and idiopathic neuropathy, unspecified |  |  |  |  |
| G730 | Myasthenic syndromes in endocrine diseases |  |  |  |  |
| H490 | Third (oculomotor) nerve palsy |  |  |  |  |
| H491 | Fourth (trochlear) nerve palsy |  |  |  |  |
| H492 | Sixth (abducent) nerve palsy |  |  |  |  |
| G590 | Diabetic mononeuropathy |  |  |  |  |
| M146 | Neuropathic arthropathy |  |  |  |  |
| M142 | Diabetic arthropathy |  |  |  |  |
| G632 | Diabetic polyneuropathy |  |  |  |  |
| G990 | Autonomic neuropathy in endocrine and metabolic disorders |  |  |  |  |
| G45 | Transient cerebral ischaemic attacks and related syndromes |  |  | Cerebrovascular | MACROVASCULAR COMPLICATION |
| I61 | Intracerebral haemorrhage |  |  |  |  |
| I63 | Cerebral infarction |  |  |  |  |
| I64 | Stroke, not specified as haemorrhage or infarction |  |  |  |  |
| I69 | Sequelae of cerebrovascular disease |  |  |  |  |
| I70 | Artherosclerosis |  |  | Cardiovascular |  |
| I24 | Other acute ischemic heart diseases |  |  |  |  |
| I20 | Angina pectoris |  |  |  |  |
| I25 | Chronic ischemic heart disease |  |  |  |  |
| I21 | Acute myocardial infarction |  |  |  |  |
| I23 | Certain current complications following acute myocardial infarction |  |  |  |  |
| I490 | Venticular fibrillation and flutter |  |  |  |  |
| I46 | Cardiac arrest |  |  |  |  |
| I48 | Atrial fibrillation and flutter |  |  |  |  |
| I22 | Subsequent myocardial infarction |  |  |  |  |
| I50 | Heart failure |  |  |  |  |
| I110 | Hypertensive heart disease with (congestive) heart failure |  |  |  |  |
| I130 | Hypertensive heart and renal disease with (congestive) heart failure |  |  |  |  |
| I71 | Aortic aneurysm and dissection |  |  |  |  |
| E105 | Insulin-dependent diabetes mellitus with peripheral circulatory complications |  |  | Peripheral vascular |  |
| E115 | Non-insulin-dependent diabetes mellitus with peripheral circulatory complications |  |  |  |  |
| E125 | Malnutrition-related diabetes mellitus with peripheral circulatory complications |  |  |  |  |
| E135 | Other specified diabetes mellitus with peripheral circulatory complications |  |  |  |  |
| E145 | Unspecified diabetes mellitus with peripheral circulatory complications |  |  |  |  |
| I792 | Peripheral angiopathy in diseases classified elsewhere |  |  |  |  |
| I724 | Aneurysm and dissection of artery of lower extremity |  |  |  |  |
| I739 | Peripheral vascular disease, unspecified |  |  |  |  |
| I743 | Embolism and thrombosis of arteries of lower extremities |  |  |  |  |
| R02 | Gangrene, not elsewhere classified |  |  |  |  |
| I702 | Artherosclerosis of arteries of extremities |  |  |  |  |
| A480 | Gas gangrene |  |  |  |  |
| L97 | Non pressure chronic ulcer of lower limb, not elsewhere classified |  |  |  |  |
| L984 | Non pressure chronic ulcer of skin, not elsewhere classified |  |  |  |  |

**Supplementary table 2**: Frequency of diabetes related complication groupings (*counts <5 are suppressed)

| Ocular | Neurological | Renal | Cerebrovascular | Cardiovascular | Peripheral | Total |
| --- | --- | --- | --- | --- | --- | --- |
|  |  |  |  |  |  | 10392 |
|  |  |  |  |  |  | 2911 |
|  |  |  |  |  |  | 2186 |
|  |  |  |  |  |  | 1822 |
|  |  |  |  |  |  | 1225 |
|  |  |  |  |  |  | 1080 |
|  |  |  |  |  |  | 932 |
|  |  |  |  |  |  | 802 |
|  |  |  |  |  |  | 707 |
|  |  |  |  |  |  | 455 |
|  |  |  |  |  |  | 400 |
|  |  |  |  |  |  | 384 |
|  |  |  |  |  |  | 346 |
|  |  |  |  |  |  | 184 |
|  |  |  |  |  |  | 180 |
|  |  |  |  |  |  | 168 |
|  |  |  |  |  |  | 155 |
|  |  |  |  |  |  | 149 |
|  |  |  |  |  |  | 130 |
|  |  |  |  |  |  | 125 |
|  |  |  |  |  |  | 106 |
|  |  |  |  |  |  | 105 |
|  |  |  |  |  |  | 101 |
|  |  |  |  |  |  | 97 |
|  |  |  |  |  |  | 93 |
|  |  |  |  |  |  | 84 |
|  |  |  |  |  |  | 76 |
|  |  |  |  |  |  | 74 |
|  |  |  |  |  |  | 69 |
|  |  |  |  |  |  | 66 |
|  |  |  |  |  |  | 63 |
|  |  |  |  |  |  | 57 |
|  |  |  |  |  |  | 53 |
|  |  |  |  |  |  | 52 |
|  |  |  |  |  |  | 49 |
|  |  |  |  |  |  | 48 |
|  |  |  |  |  |  | 45 |
|  |  |  |  |  |  | 35 |
|  |  |  |  |  |  | 32 |
|  |  |  |  |  |  | 30 |
|  |  |  |  |  |  | 25 |
|  |  |  |  |  |  | 22 |
|  |  |  |  |  |  | 21 |
|  |  |  |  |  |  | 21 |
|  |  |  |  |  |  | 18 |
|  |  |  |  |  |  | 18 |
|  |  |  |  |  |  | 15 |
|  |  |  |  |  |  | 15 |
|  |  |  |  |  |  | 13 |
|  |  |  |  |  |  | 13 |
|  |  |  |  |  |  | 13 |
|  |  |  |  |  |  | 12 |
|  |  |  |  |  |  | 11 |
|  |  |  |  |  |  | 10 |
|  |  |  |  |  |  | 9 |
|  |  |  |  |  |  | 9 |
|  |  |  |  |  |  | 8 |
|  |  |  |  |  |  | 7 |
|  |  |  |  |  |  | 5 |
|  |  |  |  |  |  | * |
|  |  |  |  |  |  | * |
|  |  |  |  |  |  | * |
|  |  |  |  |  |  | * |

**Supplementary table 3**: Results of multivariable logistic regression for 90-day post-operative mortality, restricted to individuals who underwent a major surgical resection

|  |  | Odds Ratio | P value | 95% CI | |  |
| --- | --- | --- | --- | --- | --- | --- |
| aDCSI group | No diabetes | 1.00 | (Ref. cat) |  |  |  |
|  | No complications | 1.22 | <0.01 | 1.11 | 1.35 |  |
|  | Microvascular | 1.33 | <0.01 | 1.11 | 1.58 |  |
|  | Macrovascular | 1.60 | <0.01 | 1.44 | 1.77 |  |
|  | Combined micro & macro | 2.18 | <0.01 | 1.90 | 2.51 |  |
| Age at diagnosis | <50 | 1.00 | (Ref. cat) |  |  |  |
|  | 50-59 | 1.35 | <0.01 | 1.14 | 1.60 |  |
|  | 60-69 | 2.23 | <0.01 | 1.91 | 2.60 |  |
|  | 70-79 | 4.01 | <0.01 | 3.45 | 4.65 |  |
|  | ≥80 | 8.04 | <0.01 | 6.92 | 9.34 |  |
| Sex | Male | 1.00 | (Ref. cat) |  |  |  |
|  | Female | 0.81 | <0.01 | 0.78 | 0.85 |  |
| Socioeconomic status | 1 – most affluent | 1.00 | (Ref. cat) |  |  |  |
|  | 2 | 1.16 | <0.01 | 1.08 | 1.24 |  |
|  | 3 | 1.24 | <0.01 | 1.16 | 1.33 |  |
|  | 4 | 1.41 | <0.01 | 1.31 | 1.51 |  |
|  | 5 - most deprived | 1.64 | <0.01 | 1.52 | 1.76 |  |
| Tumour site | Right colon | 1.00 | (Ref. cat) |  |  |  |
|  | Left colon | 0.88 | <0.01 | 0.83 | 0.92 |  |
|  | Colon, unspecified | 1.83 | <0.01 | 1.60 | 2.09 |  |
|  | Rectosigmoid | 0.88 | 0.02 | 0.79 | 0.98 |  |
|  | Rectum | 0.81 | <0.01 | 0.76 | 0.87 |  |
| Stage | I | 1.00 | (Ref. cat) |  |  |  |
|  | II | 1.58 | <0.01 | 1.44 | 1.74 |  |
|  | III | 1.82 | <0.01 | 1.66 | 1.99 |  |
|  | IV | 4.61 | <0.01 | 4.18 | 5.09 |  |
|  | Unknown | 4.00 | <0.01 | 3.54 | 4.52 |  |
| Urgency of surgery | Elective | 1.00 | (Ref. cat) |  |  |  |
|  | Emergency | 3.71 | <0.01 | 3.53 | 3.89 |  |
| Year of diagnosis | | 0.95 | <0.01 | 0.94 | 0.95 |  |

**Supplementary table 4**: Results of multivariable logistic regression for unplanned readmission to hospital, restricted to individuals who underwent a major surgical resection

|  |  | Odds Ratio | P value | 95% CI | |  |
| --- | --- | --- | --- | --- | --- | --- |
| aDCSI group | No diabetes | 1.00 | (Ref. cat) |  |  |  |
|  | No complications | 1.31 | <0.01 | 1.24 | 1.39 |  |
|  | Microvascular | 1.47 | <0.01 | 1.32 | 1.64 |  |
|  | Macrovascular | 1.45 | <0.01 | 1.36 | 1.55 |  |
|  | Combined micro & macro | 1.56 | <0.01 | 1.40 | 1.73 |  |
| Age at diagnosis | <50 | 1.00 | (Ref. cat) |  |  |  |
|  | 50-59 | 0.82 | <0.01 | 0.77 | 0.87 |  |
|  | 60-69 | 0.73 | <0.01 | 0.69 | 0.77 |  |
|  | 70-79 | 0.73 | <0.01 | 0.69 | 0.77 |  |
|  | ≥80 | 0.69 | <0.01 | 0.65 | 0.73 |  |
| Sex | Male | 1.00 | (Ref. cat) |  |  |  |
|  | Female | 0.89 | <0.01 | 0.87 | 0.91 |  |
| Socioeconomic status | 1 – most affluent | 1.00 | (Ref. cat) |  |  |  |
|  | 2 | 1.02 | 0.28 | 0.98 | 1.06 |  |
|  | 3 | 1.04 | 0.05 | 1.00 | 1.08 |  |
|  | 4 | 1.07 | <0.01 | 1.03 | 1.11 |  |
|  | 5 - most deprived | 1.11 | <0.01 | 1.07 | 1.16 |  |
| Tumour site | Right colon | 1.00 | (Ref. cat) |  |  |  |
|  | Left colon | 0.91 | <0.01 | 0.88 | 0.94 |  |
|  | Colon, unspecified | 1.12 | 0.04 | 1.00 | 1.26 |  |
|  | Rectosigmoid | 1.09 | 0.01 | 1.02 | 1.15 |  |
|  | Rectum | 1.59 | <0.01 | 1.54 | 1.64 |  |
| Stage | I | 1.00 | (Ref. cat) |  |  |  |
|  | II | 1.04 | 0.03 | 1.00 | 1.09 |  |
|  | III | 1.09 | <0.01 | 1.05 | 1.13 |  |
|  | IV | 1.25 | <0.01 | 1.19 | 1.31 |  |
|  | Unknown | 1.05 | 0.22 | 0.97 | 1.13 |  |
| Urgency of surgery | Elective | 1.00 | (Ref. cat) |  |  |  |
|  | Emergency | 1.02 | 0.36 | 0.98 | 1.06 |  |
| Year of diagnosis | | 1.01 | <0.01 | 1.01 | 1.02 |  |

**Supplementary table 5**: Results of multivariable logistic regression for death in hospital, restricted to individuals who underwent a major surgical resection

|  |  | Odds Ratio | P value | 95% CI | |  |
| --- | --- | --- | --- | --- | --- | --- |
| aDCSI group | No diabetes | 1.00 | (Ref. cat) |  |  |  |
|  | No complications | 1.17 | 0.03 | 1.01 | 1.35 |  |
|  | Microvascular | 1.41 | 0.01 | 1.11 | 1.80 |  |
|  | Macrovascular | 1.54 | <0.01 | 1.33 | 1.78 |  |
|  | Combined micro & macro | 2.10 | <0.01 | 1.74 | 2.54 |  |
| Age at diagnosis | <50 | 1.00 | (Ref. cat) |  |  |  |
|  | 50-59 | 1.76 | <0.01 | 1.28 | 2.41 |  |
|  | 60-69 | 3.07 | <0.01 | 2.29 | 4.11 |  |
|  | 70-79 | 6.30 | <0.01 | 4.74 | 8.37 |  |
|  | ≥80 | 12.35 | <0.01 | 9.30 | 16.40 |  |
| Sex | Male | 1.00 | (Ref. cat) |  |  |  |
|  | Female | 0.77 | <0.01 | 0.72 | 0.83 |  |
| Socioeconomic status | 1 – most affluent | 1.00 | (Ref. cat) |  |  |  |
|  | 2 | 1.22 | <0.01 | 1.10 | 1.35 |  |
|  | 3 | 1.32 | <0.01 | 1.19 | 1.47 |  |
|  | 4 | 1.51 | <0.01 | 1.35 | 1.68 |  |
|  | 5 - most deprived | 1.87 | <0.01 | 1.68 | 2.08 |  |
| Tumour site | Right colon | 1.00 | (Ref. cat) |  |  |  |
|  | Left colon | 1.07 | 0.08 | 0.99 | 1.16 |  |
|  | Colon, unspecified | 2.15 | <0.01 | 1.80 | 2.58 |  |
|  | Rectosigmoid | 1.09 | 0.29 | 0.93 | 1.27 |  |
|  | Rectum | 0.98 | 0.76 | 0.89 | 1.09 |  |
| Stage | I | 1.00 | (Ref. cat) |  |  |  |
|  | II | 1.57 | <0.01 | 1.38 | 1.78 |  |
|  | III | 1.55 | <0.01 | 1.36 | 1.76 |  |
|  | IV | 2.27 | <0.01 | 1.96 | 2.63 |  |
|  | Unknown | 4.05 | <0.01 | 3.43 | 4.78 |  |
| Urgency of surgery | Elective | 1.00 | (Ref. cat) |  |  |  |
|  | Emergency | 3.92 | <0.01 | 3.65 | 4.21 |  |
| Year of diagnosis | | 0.93 | <0.01 | 0.92 | 0.94 |  |

**Supplementary table 6**: Results of multivariable logistic regression for prolonged length of hospital stay, restricted to individuals who underwent a major surgical resection

|  |  | Odds Ratio | P value | 95% CI | |  |
| --- | --- | --- | --- | --- | --- | --- |
| aDCSI group | No diabetes | 1.00 | (Ref. cat) |  |  |  |
|  | No complications | 1.40 | <0.01 | 1.32 | 1.49 |  |
|  | Microvascular | 1.69 | <0.01 | 1.51 | 1.90 |  |
|  | Macrovascular | 1.52 | <0.01 | 1.41 | 1.63 |  |
|  | Combined micro & macro | 1.89 | <0.01 | 1.70 | 2.10 |  |
| Age at diagnosis | <50 | 1.00 | (Ref. cat) |  |  |  |
|  | 50-59 | 1.13 | 0.01 | 1.03 | 1.23 |  |
|  | 60-69 | 1.42 | <0.01 | 1.31 | 1.54 |  |
|  | 70-79 | 2.04 | <0.01 | 1.89 | 2.20 |  |
|  | ≥80 | 3.34 | <0.01 | 3.08 | 3.61 |  |
| Sex | Male | 1.00 | (Ref. cat) |  |  |  |
|  | Female | 0.83 | <0.01 | 0.80 | 0.85 |  |
| Socioeconomic status | 1 – most affluent | 1.00 | (Ref. cat) |  |  |  |
|  | 2 | 1.07 | 0.01 | 1.02 | 1.12 |  |
|  | 3 | 1.20 | <0.01 | 1.15 | 1.26 |  |
|  | 4 | 1.31 | <0.01 | 1.25 | 1.37 |  |
|  | 5 - most deprived | 1.55 | <0.01 | 1.48 | 1.62 |  |
| Tumour site | Right colon | 1.00 | (Ref. cat) |  |  |  |
|  | Left colon | 1.30 | <0.01 | 1.26 | 1.35 |  |
|  | Colon, unspecified | 1.64 | <0.01 | 1.47 | 1.83 |  |
|  | Rectosigmoid | 1.39 | <0.01 | 1.29 | 1.48 |  |
|  | Rectum | 1.91 | <0.01 | 1.84 | 1.99 |  |
| Stage | I | 1.00 | (Ref. cat) |  |  |  |
|  | II | 1.44 | <0.01 | 1.37 | 1.51 |  |
|  | III | 1.39 | <0.01 | 1.32 | 1.46 |  |
|  | IV | 1.78 | <0.01 | 1.67 | 1.89 |  |
|  | Unknown | 1.52 | <0.01 | 1.39 | 1.66 |  |
| Urgency of surgery | Elective | 1.00 | (Ref. cat) |  |  |  |
|  | Emergency | 2.50 | <0.01 | 2.40 | 2.59 |  |
| Year of diagnosis | | 0.93 | <0.01 | 0.92 | 0.93 |  |

**Supplementary figure 1**: Frequency of diabetes related complications (bars represent the number of individuals with diabetes in each subset) Intersects with a group size of ≥50 are presented

| 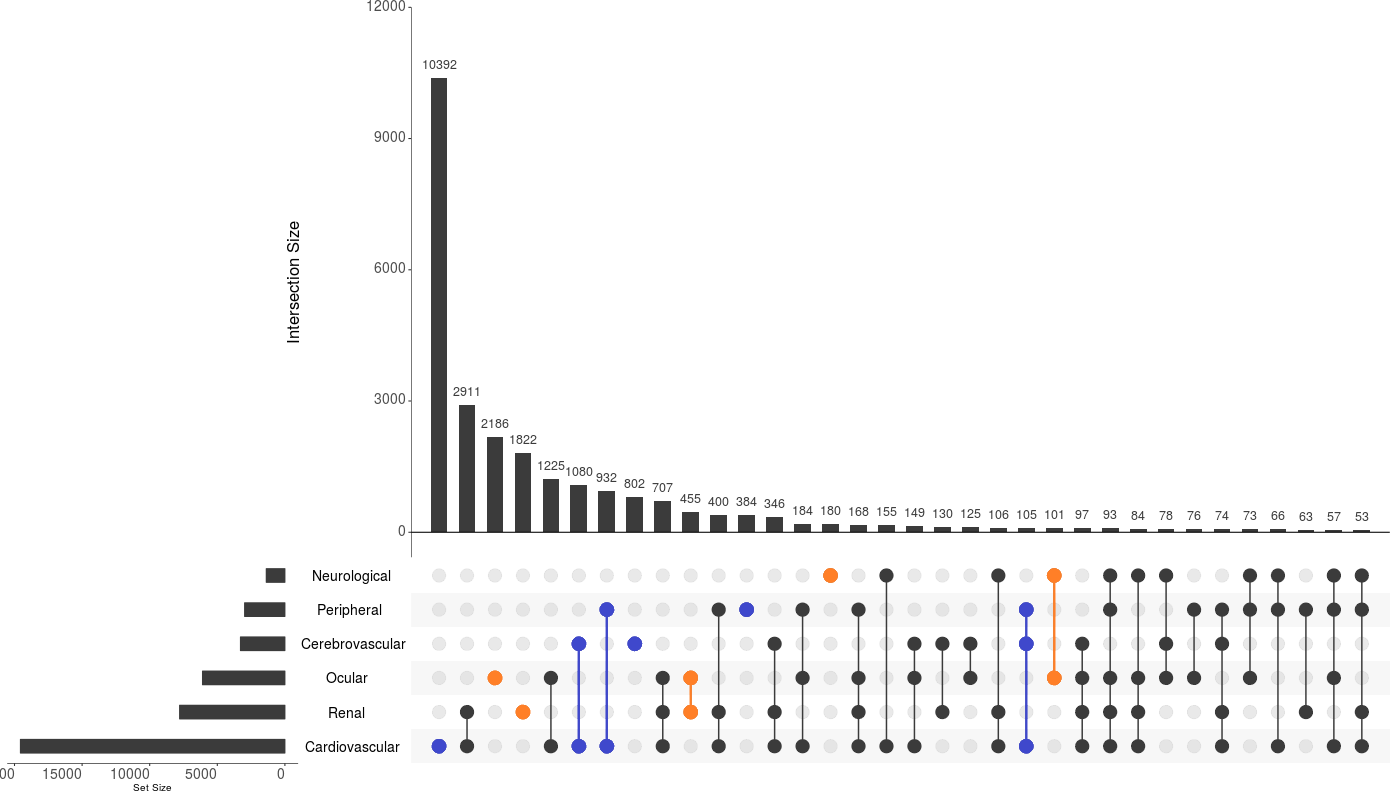 |
| --- |
| \|  \| \| \| --- \| --- \| \| Key \| \| \|  \| Microvascular complication \| \|  \| Macrovascular complication \| \|  \| Combined microvascular and macrovascular complications \| |
